# Organisation and financing of the health care systems of Bulgaria and Greece – what are the parallels?

**DOI:** 10.1186/1472-6963-5-41

**Published:** 2005-05-28

**Authors:** Nikolaos M Exadaktylos

**Affiliations:** 1 Higher Technological Educational Institute of Thessaloniki (A.T.E.I.T.), Vasilis Olgas 6, 54640, Thessaloniki, Greece

## Abstract

**Background:**

The Bulgarian and Greek Medical Care systems have been reformated the last fifteen years. The aim of this study was an examination and comparison of the Bulgarian and Greek Medical Care Systems.

**Methods:**

This study was prepared by using all the published data related to both Bulgarian and Greek Medical Care systems. Besides, personal communications with related offices such as administration offices of hospitals and Ministries of Health were made.

**Results:**

In both countries, besides the compulsory insurance there is also additional voluntary insurance which is provided by private companies. The most important difference is the family doctor (specialist in general medicine) existing in Bulgaria. Every insured person needs a 'referral form' completed by the family doctor before visiting a hospital for medical attention (except emergencies). In contrast, in Greece an insured person can directly visit any hospital without needing any forms and independent of the severity of their health problem. An important disadvantage of the Greek health system is the low number of hospitals (139), in relation to population. In contrast, there are 211 hospitals in Bulgaria, although its population is lower than in Greece.

**Conclusion:**

In both Greek and Bulgarian health systems changes must be done to solve the problems related to informal payments, limited financing, large debts, lack of appropriate investment policy, lack of an objective method for the costing of medical activities and inefficient management.

## Background

Bulgaria provided universal health care free at the point of use prior to its transition from communism in 1989. Throughout the 1990s, the Soviet-style model in central and eastern Europe that provided free health services has been subject to radical reforms [1]. The medical care system in Bulgaria, which consisted of two levels (pre-hospital and hospital), is undergoing "medical reform" which officially began in 1999 [2] (based on three laws adopted by the National Assembly of the Republic of Bulgaria: the Health Insurance Law (1998), the Law on the Professional Organizations of Physicians and Dentists (1998) and the Law on Health Care Establishments (1999), because the state funded health care system started to deteriorate with severe shortages and out of pocket expenditure reaching unaffordable levels. Reform was clearly needed, but there was no consensus on its direction, in particular, whether financing should be through taxation or social insurance.

Following the enactment of health insurance legislation in 1998, social insurance contributions (split between employer and employee) began to be deducted by employers in 1999. The amount of revenue collected initially was limited by the low tax base (given low incomes and high unemployment) and tax evasion. In 2000 the National Health Insurance Fund (NHIF) covered 13% of all public health care expenditures. It is expected that the state and municipal budgets' share of total public financing will gradually decrease over the years as the NHIF assumes an increasingly important financing role. These basic rights were developed and specified in Bulgarian Law about medical insurance were published in the Government Gazette [3].

Not much data have been published about the Greek Medical Care System. However, improvement to the Medical Care System constitutes one of the main aims of Greek Governments, as is obvious from the many reforms and modifications conducted the last twenty years [4-7].

In both Greece and Bulgaria, medical insurance can be separated into compulsory and voluntary (there are a number of non-governmental organizations in the health sector. These include organizations in Bulgaria that existed during the communist period, such as those for the blind, the deaf and the disabled. In addition, a number of newer organizations have developed, representing people with multiple sclerosis, diabetes and cancer). Voluntary medical insurance is additional to compulsory and is implemented by limited private companies holding a licence (based on the Law). This regulates the establishment of commercial and operation companies within the voluntary insurance sector. Voluntary health insurance has been limited in both Bulgaria [1] and Greece, so far taken out only by high-income groups. Voluntary health insurance can provide extra insurance (to be 'bought') on a voluntary basis by any individual. Beyond the basic package, citizens are free to buy different insurance packages on the market. Private insurance may also cover those services included in the basic package and negotiated by the National Framework Contract. Voluntary health insurance funds are also legally entitled to own hospitals and pharmacies.

## Methods

This study is the first afford to examine and compare the Bulgarian and Greek Medical Care systems. The main problem to conducting this study was the lack of much published data, especially for the Greek Medical Care System.

Therefore, all information published in journals, magazines, newspapers, Government Gazettes, books and teaching notes were used. In addition, personal communications with the administration offices of the hospitals in both countries were made. Finally, data were obtained from the Bulgarian and Greek Ministry of Health.

## Results

### Structure of the health care systems of Bulgaria and Greece

The Bulgarian health care system, in contrast to Greek system, was highly centralized and some decentralization has taken place since 1991. First, ownership of most health care facilities was devolved to locally elected municipalities from 1992. Following a 1997 amendment to the Law on Health, health facilities can become independent juridical entities. Second, the Ministry of Health decentralized much administration to the 28 regional health centres in 1995, allowing a flatter management structure. Third, there has been extensive privatization of pharmacies and physicians' practices.

As reported above, there are two main systems in both countries: private and public health system. These two systems have a tendency for approaching each other [8]. At present, private practice involves mainly dental offices and physicians' surgeries and consulting rooms, pharmacies, laboratories, and outpatient clinics and polyclinics. In addition there are inpatient health care establishments. Boonekamp [9] reported that the changes aim to adapt the new health systems to the requirements of the market. The health systems in both Greece and Bulgaria, after their re-organization, have a tendency for moving from the public to the private system.

Medical care in Bulgaria is based on the recent law and dependant on the target and the volume of the implemented medical activity, is divided into two basic groups:

a. Medical centers of hospital treatment.

b. The medical centers of non-hospital medical treatment are distinguished as follows:

1. Clinics of primary treatment:

- Individual clinics of primary medical treatment – served by a doctor specialized in general medicine.

- Corporate clinics of primary medical treatment – organized by a commercial company or a corporation served by doctors specialized in general medicine.

2. Clinics of special medical treatment:

- Individual clinic of special non-hospital medicine – served by a doctor with a recognized specialty (not general medicine).

- Individual clinic of special medical treatment – organized by a commercial company or corporation served by doctors with the same speciality (except general medicine).

- Medical center, dental center and medical-dental center – in which, at least, three doctors / dentists with different recognized specialties provide special non-hospital aid. These centers are headed by a doctor or a dentist with recognized specialty.

- Diagnostic-consultative center (multi-clinic) – in which, at least, ten doctors with different specialties work. This center has the necessary medical equipment (there is, at least, one medical-diagnostic laboratory and apparatus for imaging diagnostics). It is probably headed by a doctor with the required specialty in each case and a specialist in medical management having postgraduate studies in economics or medical information science or economics of medical care etc.

- Autonomous medical-diagnostic laboratory – in which a doctor is jointed by a specialist on medical consultations after a referral from a doctor or dentist. Depending on the specialization of the laboratory, at least, one doctor works there.

- Autonomous medical-technical laboratory – in which well – trained specialists perform special technical activities and produce special medical and auxiliary aids after a referral from a doctor or dentist. It is headed by a doctor or dentist or a specialist, depending on the specialization of the laboratory.

As reported above, political and socioeconomic impacts have caused reforms of the Greek medical care system the last twenty five years. The first reform happened in 1983 with the development of National System Health (NSH) following from five amends [4-7,10]. All the amends were based on the same rationalities and philosophy and aimed to improve and update Greek medical care [11].

Medical care is distinguished into: a) open or non – hospital care, comprising of medical services concerning the diagnosis and treatment out of hospital and b) the closed or hospital care, concerning medical and hospital services provided in the hospital for diagnosis and treatment [12].

Today, there are three levels of medical care [13]:

a. First-degree level of care – refers to reception centers, where the patient has first contact with the health system (doctor, midwife, nurse etc.). The organization of the first-degree care services are clinics, health centers, out-patients of hospitals, multi-clinics of an insurance organization. According to the manifesto of Alma Ata, as published by Dimoliatis et al., [14], the first-degree of care was based on the scientifically proved data which are easily applied with low cost. It mainly provides services of prevention, curative and recovery of patient health.

b. Second-degree level of care – refers to the medical care provided by the hospital of a region (local or prefecture). This hospital can handle basic health problems, which require hospital care. The provided services are the hospital treatment, laboratory checks for covering of hospital treatment required and general operations [13].

c. Third-degree level of care – refers to the provision of complicated or specialized health problems. The provided services are high hospitalization and curative which require well-experienced staff and well-equipped laboratories. This medical care is usually found close to population centers.

Both Greek and Bulgarian medical systems have similar hospital treatment. The basic difference is the family doctor existing in Bulgarian non-hospital treatment. In Greece, the capitulary of the family doctor system was lawed in 1997 (N.2519/97) [5], but it has not be applied yet.

In Bulgaria, the first contact of the patient with the health system is a visit to family doctor, in contrast to Greece, in which the first contact of patient with the health system is the 'out patients' department of hospital or with private doctors having a contract with the public insurance or with the polyclinics of Greek Foundation of Federal Health Insurance (Greek abbreviation, I.K.A.) or with the agricultural clinics or with the health centers. Because most of the patients visit the out patients' department of hospitals, a problem arises from the crowd of patients resulting in abnormalities in the function of hospitals. As a result of this problem it is the policy of Greek Governments to concentrate on the hospital treatments at the expense of the non-hospital treatments.

### Medical care of insured people in Bulgaria and Greece

The National Health Insurance Fund (NHIF) is an autonomous institution for compulsory health insurance that was established in accordance with Bulgarian legislation. The Health Insurance Law adopted by the Bulgarian parliament in 1998, introduced a Bismarckian type of health insurance system, with only one health insurance agency and mandatory health insurance payments deducted from personal income. Parliament decides the size of health insurance payments and each year determines the budget of the National Health Insurance Fund [15]. The medical centers of non-hospital and hospital care have individual contracts with the National Health Insurance Fund (represented by the directors of the Regional Funds) and become the providers of medical care in the system of the compulsory medical insurance.

The provided hospital care of compulsory insurance is done in the form of agreement "packages" or "clinical paths" paid by the national fund without any economic participation of the insured individual. Today, there are in total 40 clinical paths. The "clinical path" is a system of demands and behavior instructions of the different medical specialties for the hospital treatment of patients with certain diseases, included within the legal framework, which are paid by the National Fund. The "clinical path", in its own way, acts as a tool to ensure the quality of the medical activity, for the reason that the payment in hospitals is directly related to the quality of the provided medical assistance according to the relative clinical path.

Every compulsorily medically insured person can choose by his / her own personal doctor specialist in general medicine (family doctor) from a medical center (having a contract with the National Health Insurance Fund), which provides a primary non-hospital aid. The family doctor is responsible for every health problem of his patient. Depending on the severity of the health problem, the family doctor decides the treatment or refers the patient with a relative document "medical instruction consultation or performance of common cure" to the executor of the non-hospital medical aid if support from a more specialized doctor is required. The insured person can choose for himself the medical center of special non-hospital assistance with the only limitation that the chosen medical center must have a contract with the regional fund and be within the region. If there is not a medical center of required non-hospital assistance to carry out the appropriate diagnostic and therapeutic procedures within this region, the family doctor can forward the insured patient to a medical center of another region. Finally, in cases where no non-hospital foundation can provide the required treatment, the doctor-executors of this foundation prepare for the admittance of the insured to the hospital by filling a form called "Instruction for Admittance to the Hospital".

There are big economical problems in the Greek hospitals due to the low day payments from the public insurance foundations which have been in the same levels since 1993. This problem is very complicated because, if the day payments from the public insurance foundations increased, then other economical problems would be raised in the public insurance foundation. Balabanova and McKee [16] suggest that a health financing system under public control that fits well with values and population preferences is likely to improve compliance and be more sustainable. A field which must be investigated is the collaboration of the public hospitals with the private insurance companies considering the increasing number of persons with private insurance the last fifteen years [11].

Public insurance is compulsory while private insurance is not compulsory (subsidiary) in Greece. In the last few years, the percentage of private insurance has been dramatically increased as an addition to public insurance. Users pay for services not included in the packages. These can be paid for by voluntary (private) health insurance provided by private shareholding companies for additional health insurance. Citizens have the right to purchase packages of additional services from the private health insurance funds, thus guaranteeing a mixed system of public-private financing. In addition they are entitled to purchase packages offering a full range of health care services.

The basic package for primary health care contains the following services:

• ambulatory care (examination)

• surveillance, home visits, consultations

• health promotion and health prophylactics

• immunizations

• referrals for medical and diagnostic tests

• prescription of drugs, etc.

For the performance of services included in the basic package, general practitioners are paid by capitation on the basis of the number of patients on their list. In addition to the basic package of services general practitioners participate in special health programmes, called Management of Health

Priorities, including:

• maternal and infant health care

• adolescent health care

### Hospital care in Bulgaria and Greece

In contrast to the therapeutical institutions of non-hospital or pre-hospital care, these foundations have a more mutli-complex form of organization and way of practicing their medical activity.

Depending on the kind of medical care provided and the duration of treatment and stay in hospital, the medical centers of hospital care in Bulgaria are divided as follow:

a. Hospitals of active treatment – these hospitals treat patients with acute diseases, maltreatment, severe chronic diseases, conditions requiring operating treatment in a hospital, obstetric services and medical-cosmetic services.

b. Hospitals for completion of treatment and prolonged treatment – these hospitals treat patients with needs for a prolonged rehabilitation of health, people with chronic diseases requiring care.

c. Hospitals for rehabilitation – these hospitals treat people with needs for physical treatment, kinetic and psychic rehabilitation, physiotherapy, bath-therapy and sea-therapy.

d. Hospitals for completion of treatment, prolonged treatment and rehabilitation – In these hospitals, activities referring to the above b and c hospitals are carried out.

Depending on the specializations of medical activity applied in these hospitals, they are divided into specialized (with one specialization) and general (with many specializations).

Depending on the area served by a hospital, they can be divided as:

a. District – when the hospitalized patients come from one or neighboring municipalities.

b. Regional – when the hospitalized patients come from municipalities from one region.

c. Tertiary – when the hospitalized patients come from different regions.

d. National – where diagnostic and therapeutical activities and scientific research on the application of modern medical technologies are carried out (unique for the country) or duties for the processing and accomplishment of the national health policy are performed.

Finally, depending on the property status, the hospitals are divided into public (with government or municipal participation) and private.

According to evidence given by the Ministry of Health [17], the total number of hospitals in Bulgaria was 218 (included the newly-established) with a total of 47.602 beds in 2001, with a tendency of reduction at rates of 12.65% in 2002 (recorded at 30 June). The average use of beds in hospitals of active treatment with many specialties was 251 days and in the specialized hospitals of active treatment was 239 days in 2000, while for 2001 was 241 and 214 days respectively [17]. The average duration of a patient attending in a hospital for active treatment for the year 2000 was 10.2 days and for the year 2001 it was 9.6 days [17].

The different kinds of medical centers of hospital care, their establishment, organization and closing down are regulated in detail in the Bulgarian Law for the medical centers [2].

The planning and the allocation of hospitals is taken from the National Health Map as implementing of the National Health Policy.

The National Health Map of Bulgaria was sanctioned by the decision no. 688/4-11-1999 of the Council of Ministers and was published in the Government Gazette [18]. The kind, the number and the allocation of hospitals into regions are also included in the annex no. 2 [18]. From the above, it can be seen that of the 218 hospitals of Bulgaria, 127 are hospitals with many specializations for active treatment, 73 are specialized and 18 are private-owned.

The hospital care in Greece is distinguished into three categories [18]:

a. First-degree hospital care – comprises the services provided in the out-patients of a hospital, such as diagnosis and treatment of patients and emergencies cases.

b. Second-degree hospital care – refers to the admittance of a patient in the hospital for diagnosis and includes medical attendance, laboratory checking and general operations.

c. Third-degree hospital care – refers to the admittance of the patient in the hospital but in addition, it provides:

• Highly specialized knowledge

• Highly specialized skills of accession

• Highly specialized equipment

Cooperation and support of other, except the major, medical specializations [19].

Currently, Greece has 139 hospitals, of which 114 are general and the remaining 25 are specialist. Military hospitals are not included.

The hospital care is provided by different types of hospitals, distinguished as follow:

a. Depending on the extent of the rendered services, into general and specialized hospitals. The general ones have departments for treatment in more than one specialization, while the specialized have one specialization such as psychiatric, anti-cancer, obstetrics etc. The specialized hospitals cover the needs of more regions.

b. Depending on the duration of medical attendance, they are distinguished into hospitals of "acute" treatment, where the duration is less than one month and into hospitals of "chronic" diseases, where the duration is longer e.g. psychiatric, geriatric etc.

c. Depending on their legal form they are distinguished as State or L.P.P.J., Municipal, Public-Benefit, Hospitals of Insurance Organizations, and Private.

d. Depending on their geographical range and the size of the population served they are distinguished as follows:

• Local, with an area of responsibility up to 50.000 residents

• Prefectural, serving an area up to 200.000 residents. They operate in every prefecture and have departments, at least, in basic specializations providing medical training only in some specialized fields.

• Regional, operating in chair of each health region, covering the needs of people of the region. They have departments for all or most medical specialties, providing medical training for all or the most specialties contributing to the promotion of the medical research. The regional hospitals are considered as units of tertiary degree care, while the Local and Prefectural as units of secondary degree care.

e. Depending on their the type of training provided they are distinguished into:

• university hospitals

• hospitals having a limited training role

• hospitals that do not perform training work.

As in other central and eastern European countries, informal payments by patients for health care services were common in Bulgaria during the 1980s, although not officially sanctioned by the communist authorities. Such payments became increasingly common during the 1990s [1,15]. Informal payment is also a problem for the Greek Medical Care System. This problem is widely observed in the most of the Greek hospitals in which out of pocket payments are given to the hospital doctors mainly to perform operations. Balabanova and McKee [20] reportrd that informal payments stem from the low income of staff, patients seeking better treatment, acute funding of shortages and from tradition. Another problem, especially in Greece, is the creation of artificial demand for medical services. A good example is the recent estimation for both public and private medical sectors where 70% of the heart operations are performed often without necessary indications [21]. Besides, Greece is the only European country in which embryoctomy accounts for 75% of childbirths.

## Discussion

The main aim of this study was to investigate the basic differences and problems in hospital care between Greece and Bulgaria. So far, there are very few reports of both systems and a comparison could help to improve the medical services in both countries.

In both countries, besides the compulsory insurance, there is also the additional voluntary sector which is provided by private companies. The most important difference in health systems between the two countries is the family doctor (specialist in general medicine) existing in Bulgaria. Although the Greek government legislated for the family doctor with the modification of NSH in 1997 [5], the application of this was cancelled four years later [14]. A reason could be the low number of general doctors (560) [22] existing in Greece, which were not enough to cover the needs for applying this system. The reason for the low number of general doctors was that they were considered as second class doctors [23].

A basic difference between the two systems is also that, in Bulgaria, every insured person needs a 'referral form' completed by the family doctor before visiting a hospital for medical attendance (except emergencies). Therefore, by this system, patients do not get crowded in the hospitals of Bulgaria whereas in Greece patients can directly visit a hospital independent of the severity of their health problem. Other causes of the overcrowding in the Greek hospitals are the low number of available beds, the low number of hospitals (139) (in comparison to population) and the ineffectiveness of the first-degree level of care. In contrast, there are 218 hospitals in Bulgaria, although its population is much lower than in Greece. Probably, an increase in the number of hospitals in Greece could improve the medical services.

Some of the common problems for both systems are:

• Limited financing

• Big debts

• Lack of appropriate investment policy

• Lack of objective method for the costing of medical activities

• Inefficient management.

The liability insurance of Greeks in the last few years has provided of a new income for hospitals in addition to the financial support given from Government. However, in both countries problems are caused by the low sums, which are paid by the insurance organizations and from the delay of payment. Thus, the incomes from the insurance organizations can not cover the expenses of hospitals. Solutions to these problems must come from the Government. Increases in the financial support for the public medical care, reduction of expenses, and distribution of the incomes are some of the suggested solutions. Also, essential is the re-planning of the hospitalization cost and maybe, the "closed hospitalization" in the hospitals should be abolished. Especially for Greek medical care, the first-degree level of care must be improved to solve the problem of overcrowding in hospitals. In Greece, effords by the responsible ministers to improve the NSH have been ineffective.

## Conclusion

It is important to report that Bulgaria has made significant steps of progress in respect to medical care in the last few years and this is only the beginning as the medical reform has recently started (1999) and has not been completely applied. With regard to hospital care, Greece is far ahead in the technological equipment and the scientific field. However, Bulgaria is better than Greece on the viewpoint of admission to hospital. Another characteristic that should be pointed out regarding the improvement of hospital care in Bulgaria is the potential of competition among hospital foundations, according to the recent law, something that has not yet happened in Greece.

## Competing interests

The author(s) declare that they have no competing interests.

## Pre-publication history

The pre-publication history for this paper can be accessed here:


